# Genomic Mutations of Primary and Metastatic Lung Adenocarcinoma in Chinese Patients

**DOI:** 10.1155/2020/6615575

**Published:** 2020-12-08

**Authors:** Xinyu Chen, Qing Bu, Xuexin Yan, Ye Li, Qian Yu, Haiping Zheng, Liang Zhao, Yanwu Zeng, Leilei Lu, Dong Lan, Jie Ma

**Affiliations:** ^1^Department of Medical Oncology, The First Affiliated Hospital of Guangxi Medical University, Nanning, China; ^2^OrigiMed, Shanghai, China

## Abstract

Lung cancer is still the leading cause of cancer-related death worldwide. Of lung cancer, lung adenocarcinoma (LUAD) is the most common subtype. Most patients with LUAD would develop into metastasis, which limits the available treatment. Targeted therapy and immunotherapy provided options for those advanced patients. But they also broached up challenges to identify the appropriate patients. This study aims to reveal the landscapes of genomic mutations in primary and metastatic LUAD and their actionability. This study enrolled 636 patients with LUAD, of whom 85 and 551 were from patients with and without metastasis, respectively. Next-generation sequencing technology was used to retrieve their genomic information. Genomic mutations including short nucleotide variation, long variation, copy number variations, and fusions were called. The corresponding actionability was revealed. A comparison of genomic mutations and actionability between primary and metastatic LUAD was performed. In primary tumors, BRCA2 and FAT3 were significantly mutated in older patients; while in metastases, ALK and NOTCH2 were significantly mutated in younger patients. Primary tumors in male patients were significantly mutated in LRP1B and KRAS. Compared to primary tumors, metastases harbored less short nucleotide variations but more copy number variations and fusions. In metastases, chromosome 1 and chromosome 9 had less short nucleotide variations and more CNV than in primary tumors. Genomic variations of activated dendritic cells were more frequently mutated in metastases. EGFR genomic variations were negatively associated with PD-L1 and TMB. Patients with EGFR inhibitor treatment tend to have lower PD-L1 expression. The revealed discrepancy between primary and metastatic lung cancer could help guide the treatment strategies and the development of novel drugs.

## 1. Introduction

Lung cancer is still the leading cause of cancer-related death worldwide [1]. According to the cell origin, 80∼85% of lung cancer were diagnosed as nonsmall cell lung cancer (NSCLC) [2]. Of NSCLC, lung adenocarcinoma (LUAD) is at the highest frequency, accounting for approximately 80∼90% of NSCLC [3]. At diagnosis, 50∼75% of NSCLC patients were at advanced stages [4, 5]. During 2000∼2010, the five-year survival rate is less than 3∼7% for patients with advanced NSCLC [6, 7]. Within the last decade, targeted therapy and immunotherapy became widely used, having significantly improved the survival and life quality of NSCLC patients [8–10]. Meanwhile, they also broached up a crucial question about the identification of appropriate patients.

Targeted therapy and immunotherapy regularly utilize genomic mutations or derivative parameters as biomarkers for the identification of appropriate patients. Current studies have revealed mutations in LUAD including EGFR, KRAS, and ALK in different populations. Distributions of these mutations varied much with clinicopathologic factors. For example, EGFR mutations account for about 50∼62% of LUAD in Asian populations [11], but only 10% in Caucasian populations. Primary and metastatic tumors also showed discordant mutations as demonstrated in other cancers [12–14]. To date, there were only a few research studies on Chinese patients with metastases. But they either had a small sample size or concentrated on a specific metastasis or specific genes [15, 16]. Inconsistent with the mutations pattern, actionability distribution could be affected by multiple factors. For example, many passenger mutations exist, which were not responsible for the development of cancer and thus not actionable. Besides, some driver genes could not be targeted, and some untargetable mutations, such as KRAS or NRAS mutations, could be treated by blocking their downstream genes. Thus, it would also be interesting to know the discrepancy between mutation and actionability distribution in primary tumors and metastases.

In this study, we enrolled 1026 patients with NSCLC. Of them, 636 were diagnosed with LUAD. Patients with and without metastasis accounted for 13% (85/636) and 87% (551/636), respectively. Samples from those LUAD patients were collected to construct DNA libraries. They were then sequenced on Illumina NovaSeq 6000. Mutations including short nucleotide mutations, long nucleotide mutations, copy number variation, and fusion were called for each sample. Enriched signaling pathways for mutations and actionable mutations were revealed for primary tumors and metastases. Tumor mutational burden (TMB), PD-L1 expression, and their association with mutations were also investigated. We hoped that the revealed discrepancy between primary tumors and metastases could help to guide the treatment of LUAD and future medicine development.

## 2. Methods

### 2.1. Patients and Samples

This study was approved by the Research Ethics Committee of the First Affiliated Hospital of Guangxi Medical University. A total of 1026 lung cancer patients were enrolled in this study. All patients had provided written consent. Of the 1026 patients, 636 were diagnosed with LUAD. There were 85 patients having lung adenocarcinoma metastases in the brain, lymphatic node, liver, or heart. The other 551 patients failed to detect any tumor out of the primary lung adenocarcinoma. Samples were collected, fixed by 4% neutral-buffered formalin at 4°C for 24 h, and embedded in paraffin wax.

### 2.2. PD-L1 Staining

The PD-L1 staining procedure was the same as the research [17]. Briefly, formalin-fixed paraffin-embedded (FFPE) tissues were processed and stained with mouse antihuman PD-L1 antibody (Abcam, 28-8, #ab205921), 3,3'-diaminobenzidine, hematoxylin, and eosin. The percentage of cells with positive PD-L1 staining was calculated. Tumors with positive PD-L1 staining >1% were defined as PD-L1 positive.

### 2.3. Library Construction, Sequencing, and Mutation Calling

Library construction, sequencing, and mutation calling were performed by OrigiMed Corporation as previous research [17, 18]. Briefly, 620 pan-cancer genes were captured by targeting amplification. Unique molecular identifiers were added into the primers. The constructed library was sequenced on Illumina NovaSeq 6000 (Illumina, San Diego, CA) for 151 bp paired-end reads. The sequencing depth was about 2000. Raw reads were trimmed with Cutadapt (V1.18) and mapped to UCSC hg19 with BWA MEM [19]. Base quality was recalibrated with the BaseRecalibrator tool from GATK (version 3.8). Unique molecular identifiers were used to deduplicate using an in-house pipeline. Short nucleotide variation, long nucleotide variation, and copy number variation were called with Mutect2 [20], VarScan [21], Pindel [22], and CNVkit [23]. Mutations with allele frequency <0.1% were filtered out. Long nucleotide variation was defined as mutations with a length >50 bp. After calling mutations, germline mutations were filtered with mutations in the blood samples of each patient. Mutations with allele frequency >60% were filtered out for possible germline mutations. Germline mutations and populational genetic polymorphism were further filtered with multiple databases including ExAC [24], gnomAD [25], 1000 Genomes [26], and ESP6500 [27] and a custom germline mutation database prepared from a mixture of 70 normal donors. The metrics of sequence processing were listed for each sample (Supplementary Table 1).

### 2.4. Classify Patients according to Mutations in Signaling Pathways

KEGG pathways were downloaded from the official website (https://www.genome.jp). We classified each patient into mutated and wild types according to whether there was a gene in each pathway.

### 2.5. Statistical Analysis

Statistic tests including the Wilcoxon test and Fisher's exact test were used. The Wilcoxon test was used to compare tumor mutation burden (TMB). Fisher's exact test was carried out for frequency comparison between two groups. For gene-based mutation frequency comparisons, only the top 30 frequently mutated genes were performed with Fisher's exact test, and their *p* values were adjusted by the Benjamini–Hochberg procedure. *p* value <0.05 was considered significance.

## 3. Results

### 3.1. Patient Characters

This study has collected samples from 636 Chinese patients with LUAD. Of the 636 samples, 551 and 85 were primary tumors and metastases, respectively. The median age of patients without metastasis is 58, ranging from 29 to 83, and that of patients with metastasis is also 58, ranging from 33 to 79. Of the 551 patients without metastasis, 258 were female and 293 were male, and of the 85 patients with metastasis, 31 were female and 54 were male. There were 163 and 37 patients having PD-L1 expression record with primary or metastatic LUAD, respectively. Age, gender, and PD-L1 status did not show significance between patients with and without metastasis. Clinical characteristics are summarized in Table 1.

### 3.2. The Landscape of Mutations in Primary Tumors and Metastases

**Figure 1:**
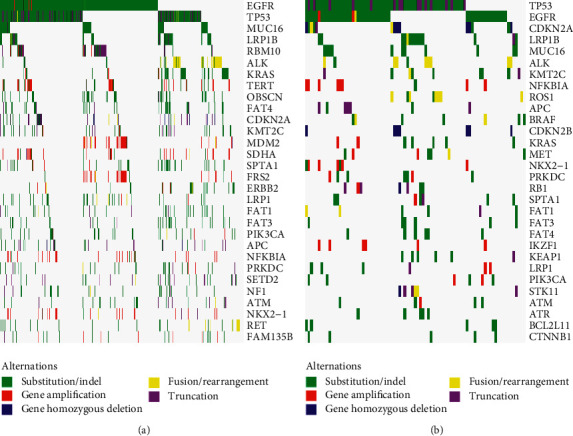
The genomic alteration profiles. (a) The genomic alteration profile in primary tumors. (b) The genomic alteration profile in patients with metastases.

Mutations including short nucleotide variation (SNV), long nucleotide variation (LONG), copy number variation (CNV), and gene-gene fusion (FUS) were inferred. Of the total 7410 mutations, there were 5662 SNV, 152 LONG, 1237 CNV, and 356 FUS. The top 30 mutated genes in primary tumors and metastases are displayed in Figures 1(a) and 1(b), respectively. The association analysis of mutations with clinical characters including age and gender was performed. In primary tumors, BRCA2 and FAT3 were significantly mutated in older patients; while in metastases, ALK and NOTCH2 were significantly mutated in younger patients. In primary tumors, LRP1B and KRAS were significantly mutated in male patients, but no significantly mutated gene was found in metastases between different genders.

### 3.3. Frequency Discrepancy between Primary Tumors and Metastases

**Figure 2:**
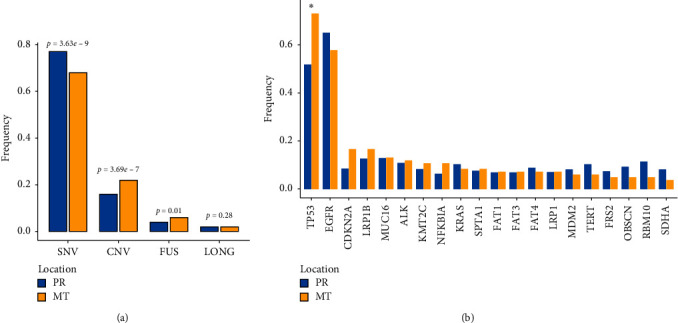
The discrepancy of genomic mutations between primary tumors and metastases. (a) Primary tumors have a higher percentage of SNV and a lower percentage of CNV and FUS than metastases. (b) The frequency of genomic mutations is compared between primary tumors and metastases ^∗^p < 0.05.

The frequency of mutations was compared between primary tumors and metastases by Fisher's exact test. In comparison to primary tumors, metastases had more FUS (*p* value = 0.01, 65/1005 vs. 127/6405) and CNV (*p* value = 3.69e − 7, 223/1005 vs. 1014/6405) but less SNV (*p* value = 3.63e − 9, 688/1005 vs. 4947/6405) (Figure 2(a)). Among the top 30 frequently mutated genes, only TP53 was found significantly mutated in metastases (*p* value<0.05, Figure 2(b)). No gene in primary tumors was found to be significantly higher mutated. Since signaling pathways were regularly used as treatment targets, we classified patients by whether they had a mutation in each signaling pathway. The top five affected signaling pathways were PI3K-AKT (93.8%), MAPK (93.3), ERBB (86.8%), FOXO (86.0%), and RAS (85.8%) signaling pathways for primary tumors and PI3K-AKT (95.4%), MAPK (95.4%), thyroid hormone (86.2%), RAP1 (86.2%), and FOXO (86.2%) signaling pathways for metastases. P53 and WNT signaling pathways were significantly higher mutated in metastases than in primary tumors (*p* value<0.05). We further checked mutations in signature genes of 22 types of immune cells and identified more mutations in signature genes of activated dendritic cells in metastases (3% vs 8.2%, *p* value = 0.03).

**Figure 3:**
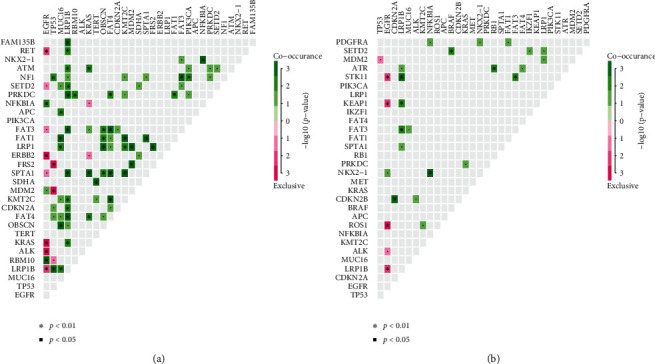
Co-occurrence of genomic alteration in primary tumors and metastases. (a) The co-occurrence relationship between genomic mutations in primary tumors is indicated with heatmap. (b) The co-occurrence relationship between genomic mutations in metastases is indicated with heatmap.

Mutation co-occurrence could provide information for drug combination therapy and medication instruction. In primary tumors, EGFR mutations were significantly exclusive of mutation in RET, ERBB2, KRAS, ALK, or LRP1B mutations and co-occurred with RBM10 and NFKBIA (*p* value < 0.01, Figure 3(a)). RET mutations significantly co-occurred with LRP1B. KRAS mutations significantly co-occurred with ATM, SPTA1, and FAT4. In metastases, EGFR mutations were significantly exclusive of mutation in STK11, KEAP1, ROS1, or LRP1B (*p* value<0.01, Figure 3(b)). STK11 significantly co-occurred with LRP1B and FAT3. KEAP1, ROS1, LRP1B, and ALK significantly co-occurred with LRP1B, KMT2C, SPTA1, and CDKN2B, respectively.

### 3.4. The Actionable Discrepancy between Primary Tumors and Metastases

**Figure 4:**
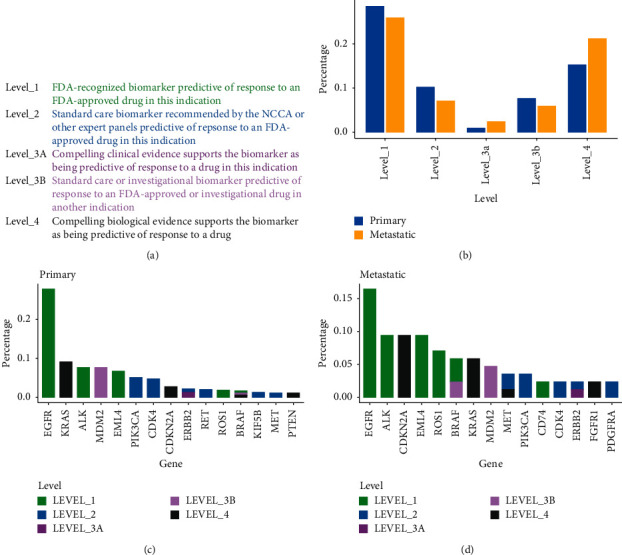
Actionability of genomic mutations in primary tumors and metastases. (a) The actionability is divided into five levels. (b) The distribution of genomic mutations at different levels is compared between primary tumors and metastases. (c) The actionable levels of genomic mutations in primary tumors are sorted by frequency. (d) The actionable levels of genomic mutations in metastases are sorted by frequency.

To evaluate the clinical significance of mutations, we classified all mutations into either nonactionable or any of five evidence levels according to the OncoKB database (Figure 4(a)). Actionable mutations had been found in 62.43% and 62.35% of primary tumors and metastases, respectively (Figure 4(b)). However, only 32.94% and 38.65% of patients with and without metastasis had standard care treatment (at evidence level 1 or level 2), respectively. Patients without metastasis had a higher percentage of standard care treatment than patients with metastasis but without a significant difference. The most actionable genes are EGFR (153/551), KRAS (50/551), MDM2 (42/551), EML4 (37/551), and PIK3CA (28/551) for primary tumors (Figure 4(c)) and EGFR (14/85), ALK (8/85), CDKN2A (8/85), EML4 (8/85), and ROS1 (6/85) for metastases (Figure 4(d)). Mutation frequency was significantly different in actionable genes between primary tumors and metastases including BRAF, EGFR, ROS1, and CDKN2A. Primary tumors had a higher percentage of actionable EGFR mutations but a lower percentage of actionable BRAF, ROS1, and CDKN2A mutations than metastases. The actionable mutations were enriched in PI3K-AKT, FOXO, and ERBB signaling pathways for patients without metastasis and in PI3K-AKT, FOXO, and MAPK signaling pathways for metastases. And primary tumors had more actionable mutations in the PI3K-AKT and ERBB signaling pathways and less in the cAMP signaling pathway than metastases.

### 3.5. Immunotherapy Biomarkers

**Figure 5:**
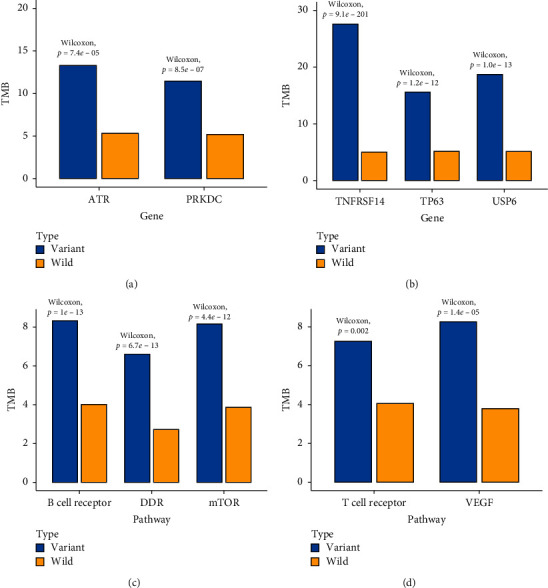
The relationship between TMB and genomic mutations. (a) Two genes (ATR and PRKDC) are associated with higher TMB in primary tumors. (b) Three genes (PNFRSF14, TP63, and USP6) are associated with higher TMB in metastases. (c) Three pathways are associated with higher TMB in primary tumors. (d) Two pathways are associated with higher TMB in metastases.

Positive cells of PD-L1 staining exceeding 1% were defined as PD-L1 positive for each tumor. TMB was found positively associated with PD-L1 status in metastases but not in primary tumors (Supplemental Figure S1). PD-L1 status and TMB were positively associated with mutations including ATR and PRKDC in primary tumors (Figure 5(a)) and TNFRSF14, TP63, and USP6 in metastases (Figure 5(b)). In all samples, PD-L1 and TMB were negatively associated with EGFR mutations. Between primary tumors and metastases, the tumor mutation burden (TMB) was not significantly different. According to the appearance of mutations in each pathway, TMB was found significantly associated with DDR, mTOR, and B cell receptor signaling pathways in primary tumors (Figure 5(c)), while in metastases, TMB was found significantly associated with the VEGF and T cell receptor signaling pathway (Figure 5(d)).

## 4. Discussion

This study aims to identify mutations in primary and metastatic LUAD and reveal their difference and the resulting clinical significance. In this study, we collected and sequenced 551 primary and 85 metastatic tumor samples. Each sample was called for SNV, LONG, CNV, and FUS. For different gender and age groups, the associated mutations were evaluated. In primary tumors, LRP1B and KRAS were significantly mutated in male patients irrespective of age. A previous study of LUAD patients with chronic obstructive pulmonary disease had revealed the prevalence of LRP1B mutations in male patients [28]. Elderly male smokers with LUAD were suggested to detect KRAS mutations [29]. In this study, patients with/without KRAS mutations showed no significant difference in age, which implied the importance of KRAS screening in male patients of all ages with LUAD. Next, we studied the association between mutations and age. In primary tumors, BRCA2 and FAT3 were significantly mutated in older patients; while in metastases, ALK and NOTCH2 were significantly mutated in younger patients. The median age of patients with ALK mutations in metastases was younger than that of other patients (49.5 vs. 60, *p* value = 0.018) while that in patients without metastasis was not significantly different (53 vs. 59, *p* value = 0.16). The median age in patients with metastasis was even smaller than that (52 years old) in another study neglecting the tumor sites of NSCLC patients [30], which suggested that ALK mutations could be more prevalent in the metastases instead of in primary tumors for younger patients.

In comparison to metastases, primary tumors had more FUS and CNV mutations but less SNV mutations. Though TMB was calculated from SNV, TMB showed no significant difference between primary tumors and metastases, which could be caused by different calculation methods. The mutation differences between primary tumors and metastases revealed that CNV and FUS mutations could play more important roles in the metastasis instead of SNV or LONG mutations. A previous comparison between primary lung adenocarcinoma and secondary metastatic brain lesion also found a higher frequency of CNV in the secondary metastatic brain lesion [31]. But no other study had revealed the higher frequency of fusions in metastases. Besides, we also revealed that metastases had less SNV in chromosome 1 but more CNV in chromosome 9. The bias in the frequency of alteration types (SNV and CNV) could originate from chromosome-level changes of the two chromosomes.

Signaling pathways were regular targets of cancer medicine. Comparison of KEGG signaling pathway enrichment showed that the P53 and WNT signaling pathways were significantly higher mutated in metastases than in primary tumors. Except the widely accepted metastasis-related gene, P53, other genes of the P53 signaling pathways were also prone to alteration in metastases (44.7% vs. 32.3%, *p* value = 0.027). WNT signaling pathways were found to play an important role in metastasis for many cancers [32]. In spite that not all mutations could be actionable, mutation distribution and actionability were highly matched at signaling pathway levels. For example, both primary tumors and metastases were enriched in PI3K-AKT, FOXO, and ERBB signaling pathways. The actionable mutations were also enriched in those signaling pathways. Except the above similarity, primary tumors and metastases also exhibited a difference in targeting signaling pathways. Primary tumors had more actionable mutations in the PI3K-AKT and ERBB signaling pathways and less in the cAMP signaling pathway than metastases, which reflected the trend of drug development in the treatment of patients with and without metastasis.

Clinical physicians regularly faced situations of lacking effective drugs. Drug combination therapy provided an alternative way to reposition approved drugs. EGFR mutations were the most frequently actionable for NSCLC, so we first studied the co-occurrence relationship between EGFR and other mutations. In primary tumors, EGFR mutations were exclusive of mutation in RET, ERBB2, KRAS, ALK, or LRP1B mutations and co-occurred with RBM10 (*p* value <0.01, Figure 3(a)). This result was different from the result of a previous study in early-stage LUAD, which found EGFR mutations co-occurred with ERBB2 mutations [33]. The contrary result could be caused by the different stages of the patients between the two studies. Metastases had different co-occurrence profiles from primary tumors. In metastases of this study, *EGFR* mutations significantly co-occurred with mutations in NOX2-1 and exclusive of mutation in STK11, KEAP1, ROS1, or LRP1B. Besides, a combination of targeted therapy and immunotherapy also triggered much interest [34]. In this scenario, the drug interaction between EGFR and PD-L1 inhibitors should take into account when performing drug combination therapy. Previous studies showed that high PD-L1 expression was associated with EGFR mutant [35, 36]. But in this study, tumors with EGFR mutations were most of negative PD-L1. The reason could be that tumors with EGFR mutations in this study were mostly treated with EGFR inhibitors which can not only inhibit EGFR activity but also downregulate PD-L1 expression as observed in a mechanistic study in cell lines [37].

A wide survey had found that the relationship between TMB and PD-L1 was not always consistent among different studies [38]. In this study, TMB was found positively associated with PD-L1 status in primary tumors but not in metastases. Thus, TMB and PD-L1 in metastases could combine to subtype patients which achieved a better outcome in the prediction of chemotherapy and targeted therapy responders [39]. Moreover, a lot of mutated pathways can potentially change TMB. And they were different between primary tumors and metastases. According to the appearance of mutations in each pathway, TMB was found significantly associated with DDR, mTOR, and B cell receptor signaling pathways in primary tumors, while in metastases, TMB was found significantly associated with the VEGF and T cell receptor signaling pathway.

## 5. Conclusion

This study revealed the landscape of mutations in primary tumors and metastases of LUAD and the corresponding actionability. The unveiled discrepancy between primary tumors and metastases could help guide the treatment strategies and the development of novel drugs.

## Figures and Tables

**Table 1:** Clinical characteristics of patients.

	Primary tumors (*N* = 551)	Metastasis (*N* = 85)	*p* value
Median age median (range)	58 (29–83)	58 (33–79)	1
Gender			
Male	304	55	0.083
Female	268	32	—

## Data Availability

The datasets generated and/or analyzed during the current study are not publicly available due to patients' information protection but are available from the corresponding author upon request.

## Supplementary Materials

Supplementary Materials Supplemental Table 1. The metrics of sequence processing for each sample. Supplemental Figure 1. The association between TMB and PD-L1 in PR and MT patients.
