# Supplementary material for: Genomic Mutations of Primary and Metastatic Lung Adenocarcinoma in Chinese Patients
Source: J Oncol. 2020 Dec 8;2020:6615575. doi: 10.1155/2020/6615575 (PMC7787720; doi:10.1155/2020/6615575)

Supplemental Figure 1. The association between TMB and PD-L1 in PR and MT patients

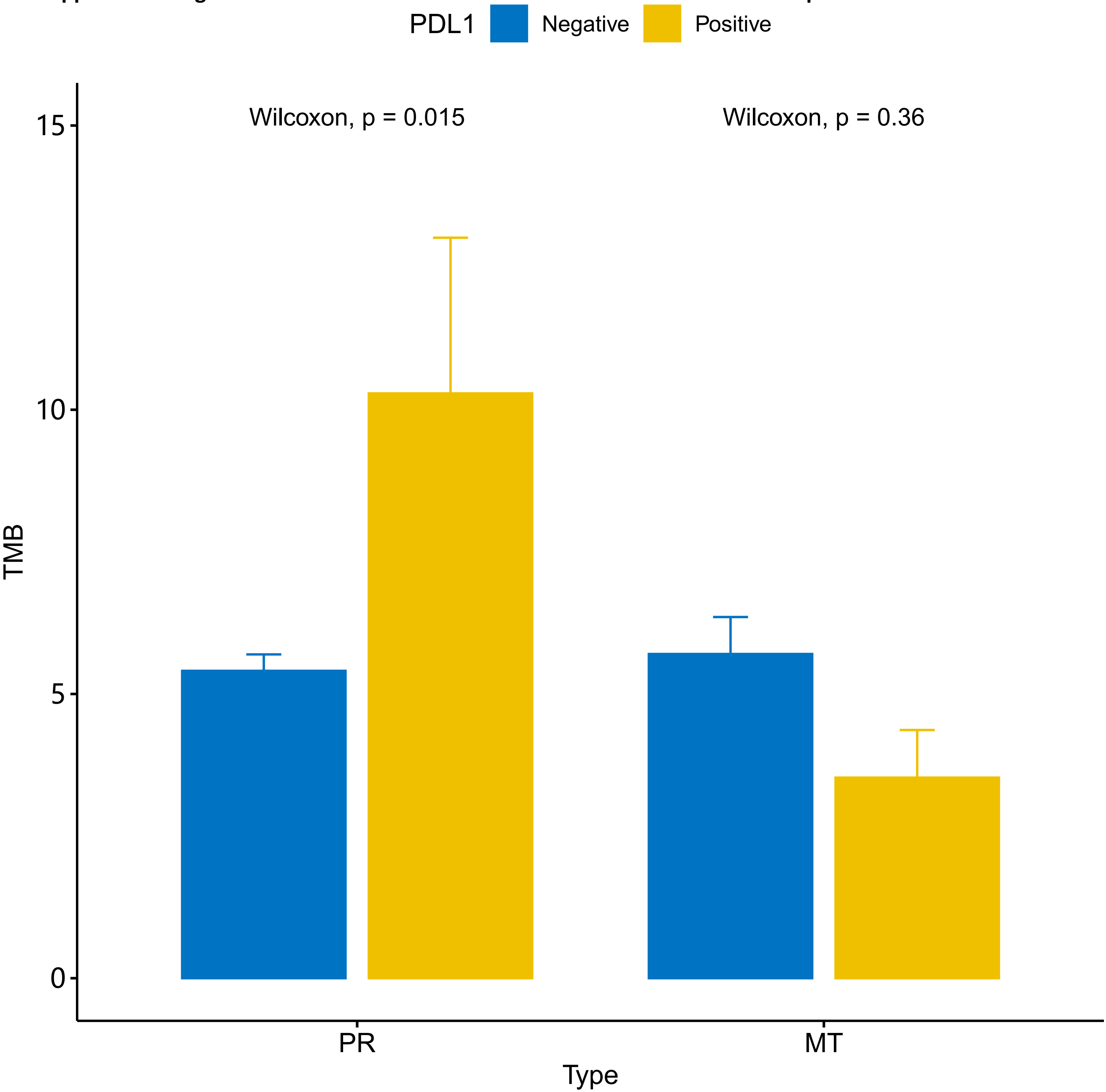

Supplement: Supplementary Materials — Supplemental Table 1. The metrics of sequence processing for each sample. Supplemental Figure 1. The association between TMB and PD-L1 in PR and MT patients. [file 6615575.f1.zip › 6615575.f1/Supplemental Figure 1.pdf]
