# Supplementary material for: Genomic Mutations of Primary and Metastatic Lung Adenocarcinoma in Chinese Patients
Source: J Oncol. 2020 Dec 8;2020:6615575. doi: 10.1155/2020/6615575 (PMC7787720; doi:10.1155/2020/6615575)
Supplement: Supplementary Materials — Supplemental Table 1. The metrics of sequence processing for each sample. Supplemental Figure 1. The association between TMB and PD-L1 in PR and MT patients. [file 6615575.f1.zip › 6615575.f1/Supplental Table 1.pdf]

Supplemental Table 1. The metrics of sequence processing for each sample

ID	PAIRS	INSERT SIZE	SEQUENCE ERROR RATE	CONTAMINATION RATIO	SEQUENCE COVERAGE	COVERAGE MEAN	COVERAGE MEDIAN	PERCENT WITH #READS > 150	MAPPED RATE
P1	26239625	205.64	0.00303	0.00045	1748.713	1182.585	1004.099	0.972	0.857
P2	32344143	233.573	0.00404	0.0167	1889.512	1262.096	1135.941	0.979	0.87
P3	32586629	234.342	0.0039	0.01414	1896.902	1266.046	1139.915	0.979	0.869
P4	20807631	233.924	0.00376	0.01127	1471.207	1130.539	1013.476	0.972	0.868
P5	45844547	240.415	0.00303	0.00191	2561.187	1199.43	1203.63	0.996	0.867
P6	42087201	230.63	0.00184	0.00263	2065.603	1325.615	1146.472	0.988	0.881
P7	31217198	185.508	0.00298	0.00208	1565.13	992.199	894.889	0.986	0.898
P8	22755222	233.797	0.00397	0.00915	1542.456	1153.86	1037.105	0.974	0.869
P9	25688595	258.674	0.00184	9e-04	1611.944	1182.256	1034.775	0.971	0.837
P10	35465633	236.652	0.00388	0.00106	1781.416	1310.108	1253.428	0.991	0.9
P11	73097528	242.2	0.00306	1e-06	2569.259	1557.779	1536.227	0.997	0.856
P12	24465573	253.138	0.00205	0.00067	1588.757	1166.866	962.932	0.952	0.849
P13	15857375	232.368	0.00524	0.02142	1292.434	1073.838	963.607	0.97	0.869
P14	40836669	234.947	0.00138	0.00045	2310.68	1533.071	1428.647	0.995	0.901
P15	37733457	260.955	0.00131	0.00207	1851.188	1388.064	1335.906	0.996	0.884
P16	66887909	247.778	0.00275	0.00514	3335.614	1313.246	1151.579	0.995	0.866
P17	39139036	232.507	0.00452	0.01218	2134.628	1337.949	1205.137	0.982	0.87
P18	14426302	246.531	0.00748	0.00444	975.865	835.315	771.212	0.956	0.873
P19	33631055	230.173	0.00321	0.00182	1666.597	1211.451	1187.393	0.991	0.9
P20	2461754	236.728	0.00323	0.0129	807.752	922.875	823.169	0.963	0.868
P21	23768126	264.244	0.00382	0.01624	1641.929	1138.08	1110.718	0.989	0.871
P22	18272974	228.473	0.00332	0.00017	1254.42	977.309	888.806	0.965	0.877
P23	38576595	245.446	0.00226	0.00028	2590.922	1658.834	1581.374	0.993	0.864
P24	25792731	276.229	0.01192	0.01575	1665.206	1146.677	1051.084	0.987	0.839
P25	26913486	252.2	0.00139	0.02607	1748.923	1218.136	1114.108	0.982	0.846
P26	46717506	244.831	0.00213	0.01917	3119.517	2012.458	1853.616	0.997	0.861
P27	26327056	226.499	0.00243	0.00064	1283.546	978.936	944.734	0.991	0.885
P28	25702786	241.618	0.00368	0.02297	1791.998	1249.21	1157.063	0.984	0.88
P29	34943641	260.57	0.00167	0.00049	1900.861	1424.202	1118.85	0.987	0.889
P30	21192866	249.255	0.00177	0.00065	1404.435	1076.541	950.89	0.981	0.874
P31	36132062	265.308	0.00384	0.00202	1814.022	1299.016	1248.512	0.993	0.899
P32	35828389	263.5	0.00282	0.00042	1997.688	1560.616	1421.36	0.996	0.89
P33	49122111	235.772	0.00309	0.0052	2495.633	1452.07	1310.207	0.987	0.87
P34	42110762	221.882	0.00212	0.00061	2424.237	1586.725	1218.637	0.981	0.908
P35	45623984	228.418	0.00176	0.00161	2054.317	1476.59	1331.652	0.989	0.817
P36	48639327	218.718	0.00159	0.00055	2794.811	1740.058	1706.526	0.997	0.905
P37	29286624	243.609	0.00198	0.00121	2153.365	1273.26	953.788	0.942	0.922
P38	40926934	204.888	0.00183	0.00197	2352.61	1565.883	1492.229	0.995	0.911
P39	25005806	217.088	0.00139	0.00042	1703.813	1074.152	989.793	0.942	0.864
P40	46552881	183.162	0.00471	0.00382	2375.302	1553.486	1368.637	0.994	0.915
P41	32676060	273.651	0.0033	0.00117	1600.374	1190.431	1149.505	0.991	0.882
P42	29464476	255.261	0.00319	0.00102	1896.517	1348.672	1220.477	0.984	0.836
P43	793247	165.6	0.00265	1e-06	2958.141	1302.158	916.597	0.924	0.821
P44	21421172	253.234	0.00301	0.01383	1466.007	1019.87	935.6	0.974	0.865
P45	58056861	207.612	0.00298	0.00722	2233.731	1472.855	704.866	0.931	0.916
P46	23045074	191.146	0.00231	0.0024	1313.936	881.666	673.014	0.92	0.912
P47	35851206	211.659	0.00231	0.00157	2064.691	1483.692	1487.955	0.995	0.918
P48	117818829	195.614	0.00268	0.00611	5958.082	1601.453	1536.25	0.997	0.869
P49	24480702	233.838	0.00183	0.00176	1543.78	1151.741	1063.613	0.974	0.824
P50	33857226	257.209	0.00282	0.00042	1896.993	1446.406	1263.013	0.992	0.896
P51	71016047	245.344	0.00226	0.00191	4036.473	2150.637	2112.493	0.998	0.89
P52	14020868	223.634	0.00312	0.00049	947.039	770.528	698.047	0.912	0.879
P53	32137873	255.757	0.00139	0.00065	1794.13	1291.527	1223.189	0.995	0.896
P54	32723279	260.071	0.003	0.00198	1602.629	1227.037	1158.458	0.996	0.885
P55	24180791	251.482	0.00148	0.00086	1182.635	868.69	833.738	0.979	0.887

P56	61326375	233.865	0.00405	0.00537	2935.536	1592.591	1442.377	0.994	0.87
P57	3093795	233.281	0.004	1e-06	1457.316	1266.531	1204.645	0.999	0.777
P58	40162318	235.605	0.00202	0.00311	1943.129	1242.161	1227.095	0.994	0.875
P59	29027843	227.814	0.00224	0.00048	1401.25	1022.659	901.711	0.962	0.862
P60	34265216	235.644	0.00429	0.02403	1956.232	1282.669	1154.455	0.979	0.869
P61	22597191	231.051	0.002	0.00044	1470.482	946.306	817.828	0.955	0.84
P62	40856432	211.94	0.00243	0.00095	1991.271	983.253	935.754	0.994	0.861
P63	27164337	343.317	0.00195	0.00056	1799.113	1284.364	890.486	0.921	0.869
P64	37984882	252.975	0.00182	0.00042	2102.371	1600.123	1047.182	0.912	0.898
P65	34387261	231.565	0.00139	0.00041	2336.542	1486.029	1407.911	0.989	0.871
P66	27316458	235.044	0.00244	5e-04	1412.922	1033.255	975.45	0.983	0.819
P67	21950787	202.295	0.00235	0.01553	1272.218	886.313	682.009	0.906	0.76
P68	37597493	268.921	0.00315	0.00046	2068.067	1478.8	1441.966	0.996	0.88
P69	35794060	211.329	0.00134	0.00175	2020.782	1317.206	1283.528	0.995	0.889
P70	14256262	235.09	0.00394	0.01369	1234.961	1057.375	947.898	0.969	0.868
P71	26402653	257.936	0.00182	0.00033	1763.299	1284.749	1157.884	0.982	0.864
P72	23191836	243.05	0.00382	0.02572	1605.721	1128.169	1086.233	0.989	0.873
P73	33793382	248.439	0.00443	0.00041	1903.745	1052.438	1048.314	0.994	0.882
P74	36753515	221.457	0.00257	0.00045	2089.141	1506.862	1377.391	0.995	0.902
P75	39015319	234.501	0.00169	0.00522	1917.645	1140.742	1017.901	0.993	0.893
P76	43434483	216.902	0.00269	0.013	2191.19	905.101	804.172	0.977	0.882
P77	36792722	250.004	0.00239	0.00167	2053.203	1385.377	1326.614	0.994	0.889
P78	30428809	261.453	0.00372	0.00078	1689.662	1231.848	1209.347	0.996	0.883
P79	23764422	247.194	0.00159	0.00059	1302.135	1046.482	751.575	0.947	0.88
P80	22888020	227.726	0.00273	0.00157	1278.767	890.25	833.195	0.98	0.883
P81	25308840	233.803	0.00362	0.02918	1634.613	1182.47	1063.438	0.975	0.869
P82	65267899	238.358	0.0024	0.00357	3077.3	1633.911	1474.96	0.995	0.87
P83	34533447	291.611	0.00146	0.00053	2165.003	1451.512	1273.37	0.987	0.818
P84	15986895	233.595	0.0039	0.01087	1295.686	1076.497	965.801	0.97	0.869
P85	19966047	205.833	0.00765	1e-06	1361.691	1026.171	706.592	0.924	0.871
P86	49030634	263.813	0.00196	0.00147	2651.337	1892.811	1675.182	0.995	0.889
P87	40822702	249.616	0.00154	0.00122	2714.245	1441.647	1274.233	0.982	0.858
P88	24468150	252.132	0.00353	0.00124	1364.811	1049.425	992.024	0.985	0.888
P89	31062462	274.638	0.00276	0.00143	1535.44	1162.83	1030.243	0.985	0.895
P90	25305636	256.84	0.00293	0.00024	1783.594	1273.94	1194.089	0.983	0.908
P91	8863795	203.9	0.00275	1e-06	2349.304	1810.767	1784.67	0.999	0.802
P92	30674492	249.46	0.00423	0.00059	1539.613	1163.006	1073.847	0.986	0.902
P93	21295833	245.525	0.00223	0.01478	1488.434	1063.697	1028.292	0.985	0.882
P94	25058500	238.58	0.00226	0.00311	1789.964	1079.049	772.288	0.908	0.912
P95	35605065	195.475	0.0029	0.00076	2016.446	1358.332	1170.901	0.986	0.897
P96	34735453	237.759	0.00232	9e-04	2291.468	1520.693	1380.169	0.991	0.852
P97	33164938	241.504	0.00138	0.01833	1917.228	1269.784	1145.118	0.979	0.87
P98	33726567	218.43	0.00309	0.0016	1772.08	1213.132	1215.036	0.996	0.826
P99	39621926	226.981	0.00232	0.00164	2240.612	1465.44	1403.172	0.996	0.899
P100	54031121	217.994	0.00256	0.0018	3092.53	1712.207	1730.484	0.996	0.893
P101	86482306	192.3	0.00358	1e-06	3445.449	1966.638	1389.232	0.98	0.882
P102	33323112	241.247	0.00231	0.00201	1868.662	1387.659	1318.709	0.994	0.896
P103	34903825	236.543	0.00255	0.01459	1980.978	1291.439	1162.574	0.98	0.869
P104	43493890	276.643	0.00167	0.00053	2907.903	2043.772	1789.609	0.998	0.876
P105	38044249	222.275	0.00136	0.00037	2130.527	1438.408	1227.169	0.989	0.891
P106	7095404	237.899	0.00276	0.01401	975.64	973.389	869.618	0.965	0.868
P107	20145749	252.326	0.00154	0.02228	1339.682	938.945	853.392	0.969	0.858
P108	20450355	224.91	0.00154	0.01233	1361.319	910.139	796.78	0.968	0.854
P109	34766127	249.462	0.00226	0.00158	1841.311	1224.544	1060.37	0.985	0.84
P110	23839715	274.246	0.00218	0.00067	1517.283	1000.946	946.683	0.983	0.827
P111	25176568	223.68	0.00195	0.00047	1641.247	1150.795	1085.973	0.977	0.843
P112	23936356	281.002	0.00161	0.02381	1514.352	1083.596	817.481	0.932	0.829
P113	33564018	288.576	0.00203	0.00048	2219.609	1712.593	1616.353	0.994	0.87

P114	26034893	248.439	0.00301	0.00031	1758.857	1413.389	1266.7	0.991	0.885
P115	62792641	197	0.00282	1e-06	2478.307	1704.719	1588.711	0.995	0.888
P116	16656159	226.659	0.00699	0.01323	1320.008	1083.761	973.803	0.97	0.868
P117	6691156	231.011	0.00567	0.01878	961.381	970.886	869.806	0.965	0.868
P118	27590613	283.956	0.00135	9e-04	1768.82	1334.011	1144.477	0.98	0.84
P119	23726772	172.719	0.00282	0.004	1157.77	715.118	648.086	0.929	0.888
P120	23214064	234.142	0.00437	0.02723	1560.037	1160.094	1042.953	0.974	0.868
P121	30785925	254.533	0.00274	0.00143	1533.277	1130.729	1034.727	0.989	0.894
P122	20712594	233.8	0.00432	0.00952	1469.007	1128.151	1012.435	0.972	0.869
P123	24624876	235.276	0.00402	0.02303	1608.226	1172.634	1053.929	0.974	0.869
P124	65516966	234.51	0.0042	0.01191	3087.866	1638.121	1481.18	0.996	0.871
P125	28785930	267.024	0.00162	0.00055	1857.085	1357.912	1085.956	0.983	0.837
P126	26812324	254.412	0.0021	0.00247	1506.879	1069.368	1043.025	0.989	0.89
P127	42156502	238.937	0.00242	0.00579	2243.589	1374.191	1239.204	0.984	0.869
P128	30030557	254.171	0.00237	0.00168	1473.07	1103.112	1027.239	0.993	0.901
P129	28741640	242.151	0.00127	0.00127	1858.283	1329.497	1093.126	0.978	0.839
P130	40226274	220.334	0.00356	0.00134	2321.265	1541.926	1532.443	0.997	0.909
P131	22148741	234.623	0.00204	0.01731	1396.568	960.289	870.014	0.965	0.823
P132	26052194	258.347	0.00138	0.00296	1721.08	1192.896	1133.527	0.983	0.854
P133	33363563	152.4	0.00182	0.00167	2016.32	1005.554	925.13	0.969	0.778
P134	49929068	230.135	0.00526	0.02092	2523.799	1461.086	1318.989	0.988	0.87
P135	40066241	196.6	0.00283	0.00019	1546.736	957.885	879.538	0.969	0.87
P136	29545085	237.833	0.00236	0.00146	1460.369	933.929	850.079	0.96	0.885
P137	61290757	199.9	0.00375	0.00657	2378.208	1453.27	1445.473	0.996	0.87
P138	37925274	225.902	0.00446	0.00088	2188.674	1444.985	1140.63	0.989	0.902
P139	31284684	232.985	0.00251	0.00037	2047.48	1485.976	1312.531	0.983	0.845
P140	40841248	233.811	0.00428	0.00693	2194.827	1358.322	1225.463	0.983	0.869
P141	45006957	269.231	0.00212	0.00046	2509.045	1662.851	1493.374	0.995	0.881
P142	34958973	231.585	0.00499	0.00181	1696.516	1190.538	1143.303	0.991	0.865
P143	71395124	209	0.00372	0.12221	2707.685	1477.83	1371.339	0.995	0.856
P144	23675192	308.464	0.0026	1e-06	1569.713	1073.027	1026.248	0.984	0.848
P145	41831512	238.412	0.00225	0.01517	2232.472	1369.805	1236.066	0.983	0.869
P146	30307264	226.253	0.00499	0.00042	1518.775	1122.243	1052.262	0.989	0.903
P147	39491191	231.865	0.00446	0.00774	2148.089	1342.946	1208.761	0.982	0.869
P148	38659501	249.305	0.00315	0.00049	2196.824	1636.417	1554.93	0.995	0.903
P149	26398610	249.964	0.00387	0.00168	1476.653	1055.734	1051.141	0.991	0.892
P150	31474441	227.714	0.01192	0.01789	2099.951	1450.308	1238.891	0.986	0.865
P151	30372562	213.472	0.00172	0.00086	2085.833	1539.903	1258.258	0.986	0.892
P152	24645065	263.555	0.00233	0.00104	1372.136	1066.364	1043.127	0.987	0.89
P153	30609882	218.37	0.0032	0.00058	1481.518	1091.165	884.112	0.974	0.878
P154	24505178	212.12	0.00371	0.00309	1620.695	1068.265	908.034	0.956	0.85
P155	36714647	300.964	0.00204	0.0019	1733.382	1308.664	1189.297	0.991	0.862
P156	24662580	242.072	0.00303	0.00041	1643.773	1282.624	1094.296	0.979	0.86
P157	33279199	242.107	0.00203	5e-04	1898.58	1426.228	1202.243	0.992	0.904
P158	18049715	236.733	0.00341	0.01381	1369.988	1099.138	985.561	0.971	0.868
P159	45381507	235.584	0.00318	0.00867	2358.971	1408.841	1272.244	0.985	0.87
P160	13566561	236.501	0.00313	0.01917	1210.478	1047.399	937.893	0.969	0.869
P161	21198506	236.056	0.00284	0.01887	1486.139	1132.853	1016.801	0.973	0.868
P162	30623501	257.558	0.00195	0.00072	2153.616	1501.758	1275.093	0.988	0.905
P163	31900138	234.141	0.0013	0.00051	1764.844	1331.834	1135.377	0.984	0.89
P164	49839990	202.024	0.00357	0.00044	2913.391	1824.878	1791.734	0.998	0.914
P165	14759504	230.421	0.00544	0.01756	1252.005	1059.168	948.739	0.969	0.868
P166	20128311	234.001	0.0035	0.00864	1445.501	1123.174	1008.076	0.972	0.868
P167	43306012	221.87	0.00399	0.01579	1524.749	933.877	943.258	0.987	0.834
P168	70668470	270.584	0.00303	0.00171	3938.658	1574.169	1339.166	0.994	0.855
P169	45716088	233.437	0.00418	0.00702	2371.555	1414.776	1275.983	0.985	0.869
P170	48807834	226.148	0.0028	0.00056	2803.736	1730.591	1723.32	0.995	0.903
P171	3403342	226.163	0.00639	1e-06	1260.663	1104.818	1009.973	0.985	0.757

P172	26916270	226.503	0.00288	0.00195	1789.477	1257.036	1222.895	0.992	0.854
P173	26534318	267.446	0.00129	0.00108	1248.641	918.969	828.49	0.963	0.869
P174	52581747	211	0.00283	1e-06	2019.761	1243.56	1198.364	0.992	0.877
P175	26550329	221.359	0.00188	0.00046	1483.393	1005.738	907.476	0.977	0.884
P176	56555558	201.398	0.00211	0.00054	2914.292	1614.769	1374.934	0.995	0.811
P177	25423610	232.305	0.0045	0.01125	1638.315	1184.705	1064.627	0.975	0.869
P178	36323131	190.862	0.00199	0.0061	2010.497	1295.278	1061.447	0.981	0.885
P179	60653636	249.92	0.00233	0.00388	3452.31	1687.815	1314.13	0.995	0.886
P180	30939270	243.226	0.00273	0.00105	1554.745	1051.128	1021.999	0.993	0.893
P181	33000264	237.208	0.00251	0.01739	1913.497	1271.827	1146.417	0.979	0.869
P182	40074198	242.68	0.00139	0.01864	2167.316	1349.239	1215.275	0.982	0.869
P183	16649199	233.571	0.00332	0.10069	1120.047	905.917	732.873	0.926	0.862
P184	25385214	216.714	0.00324	0.00059	1660.087	1257.644	1102.279	0.987	0.862
P185	42399482	257.282	0.00236	0.00174	2034.44	1332.806	1342.683	0.997	0.864
P186	35941610	190.468	0.00204	0.00437	1789.416	628.16	643.265	0.963	0.856
P187	42677404	257.17	0.00269	0.00159	2105.037	1497.428	1445.924	0.995	0.885
P188	27662047	251.793	0.00293	0.00072	1549.709	1143.472	1106.593	0.992	0.897
P189	47869120	230.989	0.00542	0.02098	2448.069	1438.553	1298.853	0.987	0.87
P190	12163393	230.976	0.00538	0.02373	1157.183	1031.123	923.969	0.968	0.869
P191	39861145	225.313	0.00202	0.00115	2647.339	1628.255	1408.735	0.994	0.848
P192	42480543	218.561	0.00192	0.00224	2395.101	1292.381	1300.709	0.997	0.888
P193	69586123	222.8	0.00327	1e-06	2903.851	1739.917	1720.277	0.997	0.884
P194	35571336	259.666	0.00225	0.02631	2317.029	1532.955	1402.901	0.993	0.843
P195	24785049	230.28	0.00483	1e-06	1710.487	1254.501	1187.476	0.995	0.881
P196	39003034	232.482	0.00475	0.01311	2126.217	1338.393	1206.272	0.982	0.869
P197	42141056	235.262	0.00271	0.0015	2415.078	1687.694	1686.897	0.996	0.909
P198	55186150	225.692	0.00249	1e-06	1911.318	1138.301	985.858	0.976	0.829
P199	44099177	223.998	0.00137	0.00475	2174.42	1420.613	1151.766	0.99	0.907
P200	48054700	226.948	0.00297	1e-06	1731.339	1254.782	1225.687	0.994	0.871
P201	34690789	255.348	0.0033	0.00125	1721.263	1201.231	1144.728	0.993	0.888
P202	43991594	242.075	0.00121	0.00024	2497.254	1786.172	1688.105	0.996	0.902
P203	38617267	234.759	0.0045	0.00154	2217.477	1596.423	1378.094	0.995	0.9
P204	35258658	287.762	0.00373	0.01313	1615.751	1201.046	1117.17	0.984	0.849
P205	24957604	252.312	0.00295	0.01282	1735.809	1045.894	1006.317	0.982	0.87
P206	40923596	191.513	0.00324	6e-04	2650.731	1226.508	1062.5	0.987	0.808
P207	50229075	215.598	0.0033	0.00537	2492.389	1228.439	1000.202	0.993	0.869
P208	35186643	238.431	0.00195	0.00986	1991.658	1293.87	1164.325	0.98	0.869
P209	55349783	218.376	0.00445	0.00056	3165.868	1852.346	1840.157	0.997	0.897
P210	42317871	263.482	0.00371	0.15936	1449.41	872.302	794.724	0.954	0.846
P211	3192883	246.07	0.00467	1e-06	1195.812	1042.45	1012.248	0.996	0.765
P212	25287878	219.683	0.00215	0.02136	1703.16	1018.886	935.676	0.975	0.861
P213	35671478	243.681	0.00156	0.00194	1786.333	1278.33	1093.914	0.991	0.904
P214	32221701	233.4	0.00212	0.00051	1727.183	1287.891	1066.251	0.979	0.856
P215	27087988	239.987	0.00235	0.0152	1782.632	1204.456	1150.895	0.991	0.854
P216	33616615	232.374	0.00444	0.00775	1932.044	1275.454	1150.753	0.979	0.87
P217	46678933	208.966	0.00357	0.00229	2691.092	1715.047	1461.166	0.996	0.905
P218	27205418	217.532	0.00127	7e-04	1776.888	1189.723	957.726	0.975	0.843
P219	24947426	277.045	0.00137	0.00207	1487.389	1191.14	1032.975	0.974	0.783
P220	35542952	204.498	0.00139	5e-04	1999.764	1305.443	1231.979	0.992	0.896
P221	32994698	256.235	0.00342	0.00143	1628.276	1268.973	1192.164	0.99	0.888
P222	24404706	227.701	0.00219	0.02057	1653.608	1179.649	957.312	0.953	0.854
P223	24302311	192.951	0.00809	0.02392	1623.682	1029.879	887.721	0.953	0.857
P224	21777268	240.075	0.00387	0.00138	1231.83	975.07	762.659	0.954	0.901
P225	29726324	237.327	0.00146	0.00054	1909.819	1291.87	1194.204	0.987	0.832
P226	25622397	238.986	0.00195	0.00064	1666.665	1110.541	1000.832	0.98	0.843
P227	51861241	251.195	0.00217	0.00046	2607.803	1454.93	1391.847	0.996	0.888
P228	51218503	306.23	0.00303	0.00038	3212.45	1409.305	1237.472	0.991	0.763
P229	1692972	233.668	0.00356	0.01982	781.196	912.444	815.904	0.963	0.868

P230	35123833	182.581	0.0026	0.00085	2294.893	1356.823	1157.227	0.984	0.841
P231	45633849	256.051	0.00155	0.00229	2247.242	1577.453	1451.425	0.996	0.885
P232	64768502	234.836	0.00349	0.01942	3060.905	1631.232	1476.751	0.995	0.87
P233	53458980	225.9	0.00324	1e-06	2031.008	1338.845	1145.532	0.986	0.875
P234	34862063	264.354	0.00271	0.00111	1924.118	1399.856	965.306	0.947	0.874
P235	21842781	261.059	0.00154	0.00049	1422.85	992.009	795.31	0.938	0.847
P236	28356856	231.31	0.00454	0.01408	1744.602	1215.803	1094.874	0.976	0.869
P237	32666846	268.452	0.002	3e-04	2098.324	1325.79	1221.842	0.992	0.832
P238	43401480	235.305	0.00414	0.02533	2286.38	1386.537	1252.102	0.984	0.869
P239	37334056	233.7	0.00245	0.0084	1392.923	904.132	759.219	0.941	0.869
P240	24744016	234.977	0.00246	0.00049	1660.155	1007.431	782.863	0.913	0.862
P241	23195741	231.949	0.00537	0.02406	1556.965	1159.595	1041.369	0.974	0.868
P242	17530298	200.123	0.00303	0.00033	1168.397	915.789	796.653	0.931	0.864
P243	15518696	235.309	0.00354	0.02796	1279.524	1068.575	956.176	0.97	0.869
P244	10230208	235.437	0.00404	0.01496	1089.291	1010.501	904.764	0.967	0.868
P245	25620934	257.347	0.00165	0.00168	1432.968	1170.331	1089.736	0.991	0.902
P246	19494560	257.865	0.00128	0.00164	1280.338	975.8	875.356	0.953	0.856
P247	44251318	221.884	0.00202	0.00101	2889.695	1740.851	1668.993	0.997	0.836
P248	30617677	256.294	0.00134	0.00046	2030.39	1446.119	1134.148	0.972	0.855
P249	49715121	270.813	0.00274	0.00039	2428.718	1690.824	1629.27	0.996	0.882
P250	23742420	233.402	0.00468	0.01292	1578.914	1164.766	1045.802	0.974	0.869
P251	22867717	240.774	0.00293	0.00056	1284.554	952.286	774.383	0.969	0.899
P252	4899235	212.8	0.00272	1e-06	1200.192	1005.461	921.271	0.99	0.76
P253	21650556	254.545	0.00246	0.00047	1421.987	939.399	790.669	0.931	0.847
P254	35639422	229.577	0.01499	0.02638	2338.737	1593.375	1498.849	0.995	0.856
P255	35815912	234.136	0.00478	0.00994	2015.342	1299.895	1171.459	0.98	0.869
P256	35833747	269.452	0.00316	0.00102	2336.48	1701.92	1595.39	0.993	0.842
P257	29717240	232.073	0.00511	0.01205	1793.78	1232.161	1107.541	0.977	0.869
P258	4847645	256.203	0.00178	0.00108	1875.957	1458.22	1165.477	0.997	0.778
P259	31985141	242.062	0.00237	0.00216	1575.148	1140.564	1000.319	0.994	0.898
P260	31419231	230.462	0.00535	0.00845	1854.954	1251.378	1126.617	0.978	0.869
P261	25501552	236.375	0.00418	0.03145	1640.515	1184.764	1062.021	0.975	0.869
P262	68995260	180.8	0.00266	1e-06	2583.244	1446.017	1248.051	0.94	0.86
P263	38744900	231.267	0.00254	0.00064	2207.422	1549.645	1347.017	0.993	0.903
P264	18012499	234.455	0.00305	0.00087	1166.159	899.124	774.419	0.947	0.847
P265	43318694	267.462	0.00236	0.00245	2088.208	1482.537	1399.427	0.994	0.885
P266	34380232	211.332	0.0028	0.00313	1672.402	1155.442	991.975	0.991	0.891
P267	46516180	235.625	0.00237	0.00135	2269.752	1299.081	1230.553	0.993	0.884
P268	30521478	218.827	0.00169	0.00152	1488.549	1055.986	1026.159	0.99	0.888
P269	31252367	220.89	0.00249	0.01459	2137.151	1382.525	1202.393	0.984	0.865
P270	47891358	273.48	0.00269	4e-04	2404.117	1525.335	1487.78	0.997	0.791
P271	35136793	315.51	0.00207	0.00187	2209.669	1252.4	1212.855	0.995	0.819
P272	36506273	257.567	0.00195	0.00102	2380.755	1655.718	1473.129	0.992	0.847
P273	43602430	238.907	0.00271	0.00049	2502.193	1771.273	1727.138	0.997	0.909
P274	61311266	240.754	0.00112	0.02271	2933.872	1592.039	1439.297	0.994	0.87
P275	35906472	209.495	0.00295	0.00072	2386.417	1491.407	1330.572	0.99	0.849
P276	40681243	211.626	0.00203	0.02787	2671.341	1394.627	1308.087	0.989	0.843
P277	32481364	257.636	0.00129	0.00196	1552.092	1019.379	847.897	0.966	0.871
P278	36752467	261.358	0.00269	0.00046	1816.585	1370.13	1326.748	0.994	0.886
P279	23908438	211.808	0.0026	0.00138	1547.528	1013.148	887.333	0.974	0.842
P280	21984717	240.534	0.00249	0.01222	1532.164	1038.648	976.53	0.973	0.882
P281	32893525	271.547	0.0031	0.00098	2375.395	1508.135	1366.871	0.982	0.914
P282	45998395	238.417	0.00289	0.01094	2381.604	1415.982	1277.412	0.986	0.869
P283	30508517	254.423	0.00282	0.00068	1694.091	1287.87	772.129	0.95	0.887
P284	31003451	232.75	0.00257	0.00041	1744.032	1366.68	1283.161	0.992	0.898
P285	31994336	259.109	0.00213	0.01813	2090.699	1448.952	1297.092	0.99	0.85
P286	21004196	245.291	0.0026	0.00101	1359.768	946.526	900.292	0.988	0.842
P287	30103650	259.728	0.00139	0.00051	2035.566	1563.409	1346.024	0.993	0.881

P288	41334369	238.464	0.00282	0.00121	2335.899	1756.598	1573.485	0.997	0.898
P289	31241370	265.953	0.00226	0.00085	2188.993	1443.496	1368.462	0.993	0.901
P290	33725599	252.763	0.00264	0.01255	2269.583	1203.54	1122.124	0.991	0.832
P291	31820434	227.147	0.00236	0.00292	1602.958	1202.795	1104.743	0.994	0.907
P292	35715787	256.515	0.00269	0.00185	1780.732	1340.356	1269.936	0.991	0.893
P293	36678166	211.421	0.00177	0.0023	2054.246	1492.576	1260.745	0.984	0.894
P294	23937459	261.906	0.00182	0.01635	1562.941	1185.535	1111.465	0.985	0.855
P295	484369	238.2	0.00337	1e-06	1990.864	1326.632	1193.01	0.99	0.875
P296	16830459	220.444	0.0026	0.00056	1063.041	744.298	616.011	0.909	0.82
P297	36550607	235.082	0.00389	0.02815	2039.152	1307.844	1178.315	0.981	0.869
P298	40091080	236.687	0.00163	0.00267	2691.238	1829.939	1435.873	0.987	0.862
P299	49527663	235.967	0.00384	0.02135	2508.344	1456.017	1313.608	0.987	0.869
P300	26252031	240.206	0.00165	0.01728	1667.653	1192.323	1073.011	0.975	0.869
P301	47480301	232.212	0.00499	0.01654	2436.512	1433.916	1294.671	0.986	0.87
P302	28357209	186.183	0.00195	0.00064	1636.629	1127.793	932.35	0.975	0.923
P303	41341226	221.14	0.00152	0.00184	2023.564	1428.541	1377.055	0.997	0.897
P304	31354416	233.339	0.00461	0.00814	1851.392	1249.2	1124.58	0.978	0.869
P305	40424847	268.253	0.00276	0.0018	2002.931	1472.923	1377.834	0.994	0.896
P306	48674723	233.329	0.00468	0.01736	2477.389	1448.169	1305.977	0.987	0.869
P307	53074469	241.397	0.0016	0.02186	2635.608	1496.265	1349.892	0.989	0.87
P308	69196408	231.5	0.00282	1e-06	2478.998	1504.244	1520.737	0.998	0.862
P309	33764049	255.467	0.00223	0.00055	1907.42	1390.742	1005.205	0.97	0.899
P310	28390807	257.752	0.00198	0.00167	2055.617	1134.994	985.544	0.941	0.902
P311	21667665	257.883	0.0039	0.01032	1500.485	1002.508	857.229	0.95	0.872
P312	32599867	240.034	0.00274	0.00184	1626.618	1220.384	1117.269	0.993	0.897
P313	55980542	226.389	0.00445	0.00193	3212.079	1952.987	1941.524	0.998	0.896
P314	26503617	264.967	0.00325	0.00126	1313.95	1018.612	976.193	0.988	0.892
P315	53495465	210.978	0.00132	0.00049	3049.417	1421.19	1412.835	0.997	0.891
P316	49940299	247.177	0.00297	1e-06	1743.375	1290.168	1249.157	0.992	0.855
P317	51087815	225.32	0.00257	0.00049	2893.314	2052.349	1993.468	0.997	0.9
P318	29986525	192.77	0.00256	0.00057	1726.847	1048.82	953.669	0.987	0.902
P319	24600322	208.408	0.00249	0.00062	1396.943	1024.3	851.306	0.978	0.903
P320	31359482	223.137	0.00256	0.0173	2191.495	1448.689	1294.463	0.985	0.88
P321	25626354	223.972	0.00316	0.00193	1684.373	1292.158	1000.78	0.963	0.847
P322	39409576	232.267	0.00429	0.00997	2143.786	1340.758	1208.296	0.982	0.869
P323	21880697	201.902	0.00256	0.01818	1565.809	1080.048	804.084	0.922	0.903
P324	45300405	253.877	0.00204	0.0021	2243.695	1593.629	1505.922	0.997	0.892
P325	35568357	230.135	0.00571	0.01739	2004.613	1298.255	1170.268	0.98	0.869
P326	48610817	238.078	0.00239	0.00548	2474.28	1445.982	1304.228	0.987	0.869
P327	27897835	229.242	0.00609	0.02032	1726.559	1209.918	1087.907	0.976	0.869
P328	48399742	238.17	0.00251	0.00657	2468.033	1444.227	1304.268	0.987	0.869
P329	33333160	215.402	0.00239	9e-04	1892.375	1100.652	1015.476	0.979	0.895
P330	29408713	258.851	0.00246	0.00266	1935.627	1460.973	1235.022	0.984	0.853
P331	20396469	207.194	0.00317	0.00109	1191.525	897.429	654.927	0.906	0.752
P332	18541444	234.518	0.00748	1e-06	1276.17	1045.925	1027.32	0.986	0.879
P333	25575924	236.644	0.00244	0.00186	1474.383	1012.677	914.99	0.983	0.911
P334	30216260	214.778	0.0013	0.00522	1694.627	1266.832	1068.049	0.982	0.898
P335	41026199	253.02	0.00168	0.00043	2719.662	1964.213	1869.76	0.996	0.852
P336	29067748	236.112	0.00203	0.00233	1641.689	1210.332	910.428	0.979	0.896
P337	27118619	272.301	0.00235	0.02999	1714.555	1241.56	1156.945	0.992	0.83
P338	25427477	247.273	0.0031	0.00099	1651.243	1144.191	1066.427	0.979	0.84
P339	15953061	233.57	0.00765	0.00052	1063.008	827.388	675.592	0.905	0.85
P340	43088925	242.007	0.0018	0.00057	2419.388	1673.39	1606.593	0.996	0.893
P341	41904530	243.601	0.00269	0.00034	2395.131	1685.5	1503.717	0.996	0.9
P342	18031725	222.954	0.00382	0.01616	1229.068	869.902	692.936	0.928	0.864
P343	25475092	250.034	0.00264	0.02122	1718.396	1205.879	1076.836	0.982	0.856
P344	30473661	210.115	0.00241	0.0019	1737.416	1113.003	1095.847	0.988	0.903
P345	72045768	193.1	0.00305	1e-06	2840.081	1633.292	1540.905	0.995	0.871

P346	35716451	198.919	0.00241	0.00179	2042.459	1155.014	1093.514	0.989	0.902
P347	64859524	269.138	0.00132	0.00037	3573.416	1690.256	1619.949	0.997	0.868
P348	24956733	233.496	0.00407	0.00752	1619.519	1178.456	1058.884	0.975	0.869
P349	28645027	231.481	0.00182	0.02186	1893.839	1299.915	1190.47	0.981	0.853
P350	27161121	226.167	0.00255	4e-04	1878.075	1451.131	1338.223	0.993	0.905
P351	19754685	235.377	0.00303	0.00047	1310.359	1051.856	924.083	0.959	0.861
P352	24354852	208.372	0.00139	0.00067	1607.442	1025.131	824.187	0.935	0.851
P353	28445148	236.59	0.00292	0.01504	1747.184	1216.984	1093.549	0.976	0.869
P354	23000887	256.169	0.00382	0.01714	1581.406	1122.159	1030.757	0.983	0.873
P355	32577698	212.865	0.00257	0.00038	1855.576	1327.157	1183.499	0.99	0.902
P356	50614076	246.056	0.00133	0.00052	3312.944	2079.732	1738.588	0.992	0.842
P357	35530711	236.737	0.00308	0.02042	2001.576	1296.472	1165.779	0.98	0.869
P358	28454776	246.845	0.00152	0.01306	1876.298	1290.353	976.958	0.947	0.857
P359	37014734	227.477	0.00174	0.00086	2531.913	1526.218	1393.118	0.98	0.87
P360	32144179	233.416	0.00293	0.00028	2293.403	1598.277	1212.861	0.977	0.91
P361	19178274	292.482	0.00192	0.00062	1202.596	927.216	812.948	0.94	0.82
P362	24918597	233.174	0.00301	8e-04	1704.14	1345.253	1195.407	0.991	0.896
P363	39991513	234.578	0.00399	0.00614	2164.396	1348.509	1215.265	0.982	0.869
P364	62233370	236.723	0.00297	1e-06	2212.419	1432.163	1373.711	0.996	0.861
P365	27328194	241.116	0.00809	0.01358	1799.616	1093.804	852.447	0.953	0.84
P366	56406093	246.568	0.00209	0.00305	4098.556	1979.588	1627.562	0.99	0.915
P367	22915984	222.284	0.00189	0.00233	1302.909	937.181	903.485	0.984	0.902
P368	21902799	255.686	0.00154	0.00048	1443.257	1041.869	885.38	0.961	0.857
P369	33998550	273.92	0.00153	0.00209	1624.032	1215.827	1176.516	0.99	0.882
P370	41524942	239.795	0.00223	0.01005	2220.281	1366.431	1232.942	0.983	0.869
P371	54737548	231.745	0.00502	0.00449	2698.297	1517.149	1371.011	0.99	0.87
P372	21729826	231.514	0.00499	0.01113	1504.466	1142.771	1027.01	0.973	0.869
P373	29932416	212.884	0.00273	0.00146	1624.377	988.993	967.719	0.992	0.842
P374	39983508	259.089	0.00184	0.00177	1947.961	1442.476	1347.174	0.994	0.878
P375	46160376	231.62	0.00141	0.00085	2632.043	1727.18	1519.133	0.992	0.894
P376	46449908	247.8	0.00325	0.01195	1277.171	877.015	786.958	0.971	0.834
P377	31953287	237.487	0.00235	0.00047	2106.038	1518.356	1208.989	0.98	0.848
P378	47817084	168.43	0.00748	0.00685	3337.438	1637.468	1410.659	0.993	0.873
P379	48067419	227.484	0.00652	0.01417	2457.599	1439.244	1298.446	0.987	0.869
P380	34536438	254.061	0.00176	0.00043	1941.279	1344.09	1306.04	0.996	0.897
P381	34918186	238.403	0.00281	8e-04	2295.873	1596.69	1281.157	0.986	0.849
P382	39806843	248.7	0.00208	0.00368	1942.74	1481.892	1269.48	0.989	0.88
P383	36630783	218.487	0.00293	0.00042	2041.03	1361.233	1311.047	0.996	0.886
P384	20333009	248.472	0.00639	0.00856	1381.083	1101.724	1073.182	0.984	0.871
P385	17485056	203.872	0.00154	0.00133	1123.549	681.437	633.233	0.922	0.835
P386	33994861	231.321	0.00183	0.00114	1938.339	1387.699	1382.241	0.995	0.907
P387	42935668	211	0.00333	1e-06	1855.152	1398.257	1230.716	0.985	0.903
P388	35680657	237.174	0.00299	0.00689	2007.799	1299.205	1169.061	0.98	0.869
P389	36438427	234.924	0.00402	0.01491	2035.844	1307.401	1177.779	0.981	0.869
P390	33431149	258.507	0.00324	0.00077	2160.714	1643.069	1591.582	0.996	0.85
P391	13884851	223.024	0.0051	1e-06	966.644	792.827	700.176	0.949	0.888
P392	42032326	216.799	0.00244	0.00111	2104.124	1352.575	1289.615	0.995	0.894
P393	36476789	233.85	0.00177	0.00175	2052.084	1404.86	1343.51	0.993	0.891
P394	34910327	256.872	0.00267	0.00044	1947.072	1287.624	1184.789	0.99	0.884
P395	38816999	240.5	0.00361	0.00219	1888.424	1350.932	1309.601	0.997	0.874
P396	16748292	233.844	0.00403	0.02324	1323.074	1085.034	974.96	0.971	0.869
P397	48782309	238.41	0.00243	0.01066	2482.968	1448.429	1306.285	0.987	0.869
P398	51816553	236.238	0.00286	0.02348	2592.775	1483.369	1340.665	0.989	0.87
P399	32641375	232.818	0.00483	0.02482	1899.848	1265.061	1137.965	0.979	0.869
P400	53222285	210.745	0.00203	0.02171	3488.085	1674.867	1571.775	0.995	0.83
P401	24984546	197.358	0.00333	0.00082	1398.073	965.161	814.578	0.982	0.897
P402	42338326	229.062	0.00204	0.00257	2084.063	1507.519	1082.709	0.986	0.882
P403	58357677	273.848	0.00241	0.00079	2796.365	1831.381	1780.74	0.998	0.882

P404	37564552	235.827	0.0026	0.00076	2472.03	1607.525	1553.013	0.998	0.848
P405	20739894	221.816	0.00329	0.01876	1345.737	990.345	770.26	0.906	0.848
P406	23032725	236.299	0.00219	0.01665	1638.712	1074.028	915.693	0.959	0.889
P407	33619295	257.828	0.00297	0.00118	2199.61	1470.556	1342.626	0.991	0.843
P408	18983759	237.194	0.00319	0.01996	1405.99	1111.148	997.732	0.972	0.869
P409	29533662	254.411	0.00318	0.00076	1853.119	1383.725	1316.322	0.996	0.843
P410	25593701	279.372	0.00145	0.00043	1658.125	1159.107	893.169	0.937	0.845
P411	54977256	265.428	0.00256	0.00145	2634.001	1796.869	1766.531	0.997	0.876
P412	51160316	234.034	0.00407	0.00698	2566.207	1475.23	1331.481	0.988	0.869
P413	68768284	220.2	0.00327	1e-06	2899.111	1762.77	1759.643	0.998	0.887
P414	34637511	249.603	0.0045	0.00156	1949.533	1471.839	1441.965	0.997	0.895
P415	53661669	245.523	0.00414	0.00037	3032.829	2195.504	2181.098	0.998	0.9
P416	48174498	235.325	0.00436	0.00925	2459.465	1442.102	1301.992	0.987	0.87
P417	28445215	258.443	0.00349	0.00215	1359.942	963.042	944.217	0.99	0.868
P418	41184033	235.102	0.00499	0.00037	2029.937	1507.055	1460.162	0.994	0.883
P419	6531322	199.6	0.00272	1e-06	1663.258	1377.377	1212.932	0.995	0.779
P420	19325180	231.786	0.00492	0.02621	1416.283	1115.494	1001.835	0.972	0.869
P421	33362723	255.449	0.00495	0.16045	1138.927	798.378	718.572	0.921	0.847
P422	6293518	200	0.00333	1e-06	1587.769	1266.202	1213.108	0.994	0.767
P423	40681510	238.145	0.00202	0.00281	2021.786	1212.052	1022.791	0.985	0.899
P424	34560373	266.715	0.00342	0.0014	1693.219	1320.472	1231.415	0.991	0.882
P425	86869093	214.713	0.0013	0.00605	4396.67	1209.637	1192.057	0.99	0.864
P426	54236220	238.191	0.00279	0.00113	3028.433	1496.915	1366.921	0.992	0.866
P427	32487645	244.768	0.00131	0.0019	1563.149	1171.13	984.815	0.984	0.865
P428	44028408	224.357	0.00138	0.00027	2504.289	1765.151	1727.571	0.997	0.905
P429	43735632	247.043	0.00279	0.00225	2428.649	1245.656	1038.532	0.981	0.866
P430	26818122	231.194	0.00195	0.02538	1776.283	1251.994	937.171	0.963	0.853
P431	28320145	171.241	0.00161	0.00878	1412.86	824.113	784.361	0.966	0.771
P432	22212470	244.361	0.00301	0.01493	1545.285	1100.047	977.485	0.974	0.88
P433	44634276	236.068	0.00324	0.02554	2333.13	1401.879	1265.415	0.985	0.869
P434	43879400	232.837	0.00284	0.00198	2176.843	1541.362	1374.531	0.993	0.885
P435	17235719	232.175	0.00303	0.00034	1152.473	921.096	873.711	0.963	0.864
P436	6420717	234.1	0.00357	0.1176	1543.92	1274.712	1257.107	0.999	0.756
P437	48841339	202.6	0.00554	1e-06	1801.279	1165.593	1119.81	0.991	0.871
P438	29115657	257.977	0.00198	0.00152	2103.145	1198.386	996.141	0.944	0.902
P439	68631941	202.9	0.00372	0.10127	2573.658	1422.238	1141.794	0.976	0.853
P440	42294671	244.586	0.00287	0.00044	2084.141	1368.072	1266.602	0.992	0.879
P441	26369010	247.323	0.00127	0.00081	1730.105	1210.565	1144.996	0.985	0.849
P442	20910421	278.999	0.00159	4e-04	1151.093	989.551	961.074	0.985	0.891
P443	46732294	234.277	0.00306	4e-04	2345.662	1490.369	1457.068	0.997	0.888
P444	32854439	226.898	0.00271	0.00237	1905.7	1345.581	1029.667	0.977	0.912
P445	46680675	226.504	0.00317	0.00049	3107.585	1994.446	1508.858	0.991	0.848
P446	18038940	218.822	0.00172	0.00104	1173.599	912.214	581.251	0.904	0.858
P447	49805109	248.055	0.00138	0.00057	3548.067	2197.849	1884.046	0.994	0.91
P448	20495009	259.589	0.00148	0.00127	994.806	722.036	680.273	0.93	0.884
P449	41630422	256.857	0.00224	0.00209	1988.799	1417.674	1066.667	0.977	0.854
P450	40648167	327.581	0.00215	0.03015	2558.38	1140.63	1108.328	0.994	0.794
P451	22160205	240.954	0.00487	1e-06	1512.855	1225.874	1151.139	0.993	0.873
P452	24817675	235.375	0.00327	0.00161	1203.444	895.244	794.266	0.978	0.893
P453	27483032	235.47	0.00452	0.01319	1712.353	1206.701	1085.704	0.976	0.869
P454	29875808	209.815	0.00154	0.02204	1976.166	1105.704	1062.839	0.993	0.84
P455	29305723	254.786	0.00204	0.0014	2073.726	1321.753	1195.029	0.984	0.907
P456	52076324	259.646	0.00146	0.00049	2922.088	2109.028	1936.236	0.998	0.899
P457	53307795	255.866	0.00319	0.00063	2926.121	1733.321	1606.233	0.997	0.869
P458	40195296	245.388	0.00303	0.00053	2282.354	1740.607	1107.64	0.981	0.9
P459	49560787	243.542	0.00259	1e-06	1732.188	977.134	941.693	0.979	0.849
P460	34657172	235.474	0.00379	0.0121	1970.55	1287.085	1157.724	0.98	0.869
P461	22679855	253.602	0.00246	0.00037	1463.939	1006.887	929.014	0.963	0.835

P462	29272066	233.936	0.00233	0.00247	1613.438	1207.598	1111.652	0.99	0.879
P463	2493384	231.285	0.00621	0.02334	807.538	922.297	822.963	0.963	0.868
P464	30305770	316.704	0.00289	0.00253	1398.094	1012.95	969.665	0.985	0.853
P465	22434736	232.588	0.00475	0.01176	1530.785	1147.978	1031.292	0.973	0.869
P466	15980031	255.406	0.00249	0.01246	1125.963	794.812	705.173	0.92	0.89
P467	25429152	265.68	0.00293	0.00084	1813.463	1224.19	1140.239	0.985	0.912
P468	53575819	242.53	0.00257	0.00812	2995.323	1511.088	1399.811	0.996	0.864
P469	56796634	190.8	0.00338	0.00884	1979.255	1190.923	1067.493	0.931	0.89
P470	32880918	248.4	0.00204	0.00184	2354.609	1411.671	1294.258	0.986	0.914
P471	52994435	232.581	0.00487	0.01382	2634.968	1494.791	1350.062	0.989	0.87
P472	27545092	229.622	0.00372	0.00035	1558.163	1226.881	1160.507	0.992	0.9
P473	28188693	298.567	0.00162	0.00256	2000.572	1305.78	1204.06	0.971	0.905
P474	37861814	239.935	0.0017	5e-04	2159.179	1494.827	1415.086	0.993	0.908
P475	37308544	221.686	0.00198	0.00139	2035.419	883.661	840.653	0.993	0.817
P476	47723274	166.664	0.00207	0.00217	2965.843	1354.148	1141.548	0.98	0.788
P477	24155822	240.868	0.00293	3e-04	1734.567	1240.089	1160.742	0.983	0.919
P478	40235598	227.802	0.00253	0.00183	2001.303	1403.006	1383.226	0.995	0.903
P479	52217601	232.121	0.00464	0.0055	2605.606	1488.607	1342.661	0.989	0.869
P480	30726284	263.583	0.00335	0.02162	1997.034	1360.247	1035.153	0.972	0.838
P481	16185426	235.63	0.00346	1e-06	1104.446	963.451	899.21	0.981	0.88
P482	40517502	257.586	0.00162	0.00057	2921.784	1841.788	1722.181	0.989	0.912
P483	38150478	212.8	0.00288	1e-06	1628.777	1221.416	1165.447	0.988	0.896
P484	55657065	210.2	0.00267	0.00685	2040.464	1232.614	1218.312	0.99	0.872
P485	57503419	238.18	0.00246	0.00931	2796.577	1547.298	1399.127	0.992	0.87
P486	53504471	242.063	0.00168	0.00059	2509.334	1390.138	1417.194	0.993	0.839
P487	32897184	185.406	0.00139	0.00232	2200.463	1261.445	1011.354	0.961	0.852
P488	21643016	263.41	0.00294	0.03102	1323.162	934.798	870.981	0.971	0.79
P489	24983024	248.049	0.00295	0.01116	1717.863	1087.228	968.492	0.965	0.861
P490	27080161	218.654	0.00325	0.00044	1392.127	1012.433	911.021	0.975	0.918
P491	27547568	273.948	0.00182	0.00027	1806.25	1317.837	1148.349	0.978	0.851
P492	3567299	217.032	0.00565	1e-06	1322.955	1137.853	1038.392	0.982	0.756
P493	19002146	238.97	0.00467	1e-06	1313.765	1070.096	980.348	0.976	0.887
P494	28062095	265.36	0.00295	0.01079	1937.629	1155.051	1136.316	0.986	0.868
P495	15781163	228.628	0.00559	0.01329	1291.028	1076.429	964.968	0.97	0.868
P496	26218618	270.502	0.00148	0.00144	1261.869	865.867	816.581	0.975	0.876
P497	30505411	242.686	0.00317	0.00058	1987.661	1406.298	1029.9	0.965	0.843
P498	44882027	246.816	0.00209	0.00115	2420.216	1615.422	1604.432	0.997	0.848
P499	35569600	285.375	0.003	0.0022	1725.644	1320.993	1242.306	0.994	0.878
P500	36938061	245.583	0.00275	0.0019	1842.548	1333.493	1079.432	0.983	0.898
P501	23153241	235.486	0.00433	0.01947	1554.879	1158.081	1039.884	0.974	0.868
P502	25928624	253.857	0.00138	0.0019	1631.568	1146.558	947.673	0.961	0.813
P503	23223132	291.569	0.00256	0.02069	1562.678	1195.253	1097.986	0.967	0.857
P504	30808382	235.197	0.00252	0.00189	2183.908	1347.521	1057.838	0.971	0.906
P505	33832990	213.748	0.00239	0.00188	1920.946	1116.429	1106.186	0.991	0.898
P506	38241750	238.365	0.0024	0.0143	2101.614	1328.556	1197.716	0.982	0.869
P507	67652586	213.918	0.00226	0.01516	5072.089	1229.675	816.068	0.902	0.896
P508	32927794	258.343	0.00276	0.00064	1618.598	1189.937	1039.571	0.986	0.886
P509	45628182	237.289	0.00245	0.00728	2366.889	1412.22	1275.142	0.985	0.869
P510	32042078	263.281	0.00134	0.00038	2062.083	1556.132	1332.242	0.985	0.84
P511	26040848	215.095	0.00201	0.00067	1263.984	881.4	735.47	0.96	0.897
P512	92492018	256.164	0.00153	0.00138	4544.657	703.658	704.008	0.994	0.787
P513	32267411	228.318	0.00169	0.00156	1562.688	1002.414	975.151	0.992	0.876
P514	52349085	223.5	0.00292	1e-06	1986.899	1230.976	1213.376	0.994	0.872
P515	16106322	221.105	0.0051	1e-06	1123.682	912.989	814.463	0.958	0.89
P516	41441585	281.436	0.00182	0.00075	2302.153	1642.354	1613.306	0.996	0.89
P517	40554150	233.648	0.00384	0.02084	2182.242	1354.175	1220.061	0.983	0.869
P518	89860559	210.719	0.00231	0.00406	5180.231	767.501	765.462	0.996	0.832
P519	20075718	230.765	0.00136	5e-04	1114.307	829.552	707.243	0.962	0.894

P520	23706355	243.562	0.00443	0.00166	1346.536	932.819	910.9	0.98	0.9
P521	44107956	224.907	0.00248	0.00047	2145.049	1400.404	1036.09	0.983	0.888
P522	44507971	251.151	0.00269	0.00049	2222.959	1557.941	1463.619	0.995	0.896
P523	31000113	209.3	0.00275	0.07311	1154.351	854.905	761.41	0.945	0.861
P524	23567067	231.027	0.00231	0.01623	1554.894	1109.306	1063.132	0.984	0.861
P525	52929247	205.157	0.0022	0.00072	3031.598	1857.34	1530.99	0.996	0.906
P526	44920058	238.08	0.00269	0.01474	2343.2	1405.478	1267.378	0.985	0.869
P527	26150267	180.75	0.00699	0.00039	1792.956	1240.254	1048.933	0.973	0.87
P528	20299722	232.964	0.00434	0.01382	1451.603	1125.377	1009.996	0.972	0.868
P529	26805060	231.906	0.00463	0.03066	1687.995	1200.054	1077.262	0.976	0.869
P530	29706092	238.548	0.00357	0.00121	1661.122	1206.886	1062.851	0.99	0.891
P531	34525886	233.664	0.00443	0.02078	1967.866	1287.909	1161.716	0.98	0.869
P532	31228853	224.082	0.00209	0.00048	1782.276	1266.055	1232.769	0.996	0.905
P533	27823323	225.139	0.0021	0.00226	1593.1	1050.113	856.019	0.968	0.902
P534	51111613	235.8	0.00377	1e-06	1823.935	1162.755	1123.646	0.94	0.87
P535	23840912	259.035	0.00249	0.0172	1639.878	1128.639	968.27	0.968	0.872
P536	48388938	228.97	0.00637	0.00751	2468.38	1445.215	1303.138	0.987	0.869
P537	26996431	275.73	0.00125	6e-04	1785.13	1328.009	1072.331	0.986	0.871
P538	49935762	224.4	0.00239	0.00218	2207.219	924.473	922.553	0.981	0.75
P539	39879163	273.155	0.00139	0.00064	2198.811	1519.348	1450.721	0.995	0.883
P540	41543894	233.984	0.00419	0.00956	2220.296	1366.921	1232.696	0.983	0.869
P541	58564531	241.144	0.00133	0.00465	2834.835	1558.049	1409.067	0.992	0.87
P542	40368520	240.393	0.00156	0.01579	2178.293	1353.936	1220.291	0.983	0.869
P543	36375915	204.5	0.00375	0.10164	1420.652	1027.607	928.909	0.973	0.887
P544	62593910	240.271	0.00274	0.00083	3094.88	1713.833	1723.999	0.998	0.88
P545	28340139	255.549	0.00164	0.00234	1394.689	1037.116	955.001	0.974	0.903
P546	44606464	230.868	0.00547	0.01403	2331.016	1400.498	1263.47	0.985	0.87
P547	72439929	265.615	0.00176	0.01262	4042.378	903.833	900.023	0.994	0.823
P548	31813811	210.496	0.00345	0.00066	1833.516	1329.646	1219.02	0.991	0.918
P549	34179750	239.48	0.00273	0.0027	1897.506	1224.479	1069.035	0.983	0.88
P550	49433387	242.903	0.00574	1e-06	1662.143	1177.185	1119.592	0.985	0.826
P551	45645966	240.249	0.00279	0.00096	2545.675	1286.249	1046.506	0.974	0.868
P552	23789745	228.3	0.00565	0.00538	1645.996	1258.496	1222.517	0.991	0.883
P553	68931762	245.742	0.00151	0.00185	3372.689	1979.7	1959.346	0.998	0.889
P554	47481483	262.582	0.002	0.0028	3072.057	1931.773	1887.047	0.996	0.836
P555	24718614	219.768	0.00809	0.0141	1621.235	1063.235	911.689	0.954	0.841
P556	50839275	235.559	0.00361	0.00042	2587.859	1764.723	1665.78	0.997	0.908
P557	65951212	229.1	0.00605	1e-06	2356.259	1412.695	1373.768	0.997	0.867
P558	44580033	220.116	0.00232	0.00429	2091.427	1283.799	826.078	0.944	0.887
P559	49641686	235.885	0.00313	0.0114	2512.593	1459.977	1317.862	0.988	0.869
P560	46530004	239.102	0.00236	0.00623	2400.205	1420.711	1280.392	0.986	0.869
P561	28996231	244.739	0.00235	0.03043	1875.149	1344.485	1020.901	0.97	0.84
P562	26700691	230.773	0.00256	0.01512	1862.244	1263.092	1104.171	0.963	0.884
P563	27422772	210.223	0.00303	0.00041	1683.249	1269.142	1215.863	0.989	0.79
P564	17796008	230.48	0.00295	0.01339	1232.329	834.984	682.12	0.921	0.874
P565	30863899	259.089	0.00293	0.00195	2170.193	1554.501	1435.846	0.994	0.905
P566	42463082	233.259	0.0045	0.00626	2254.09	1375.784	1241.587	0.984	0.869
P567	23464208	244.697	0.00324	0.01758	1658.37	1113.001	1078.944	0.982	0.887
P568	34962287	231.518	0.0021	0.00277	2032.064	1560.462	1435.855	0.995	0.918
P569	36460398	232.168	0.00469	0.01259	2036.557	1308.821	1178.66	0.981	0.869
P570	27359021	197.823	0.00172	0.00683	1896.44	1373.466	1074.201	0.982	0.904
P571	29779109	230.191	0.00591	0.00795	1796.845	1234.285	1109.992	0.977	0.869
P572	32207999	238.788	0.003	0.00281	1600.087	1194.157	1082.957	0.987	0.897
P573	51281716	217.1	0.00296	1e-06	1977.452	1184.582	1114.162	0.985	0.882
P574	64055475	241.8	0.00351	1e-06	2260.266	1406.102	1379.475	0.996	0.862
P575	57594002	245.676	0.00279	0.00231	3147.49	1477.07	1318.96	0.993	0.853
P576	17963799	244.602	0.00223	0.01453	1216.738	868.905	773.866	0.939	0.862
P577	30019431	227.443	0.00182	0.00052	1680.634	1300.503	1137.314	0.986	0.893

P578	28023510	226.05	0.00146	0.00092	1810.68	1235.804	1059.679	0.981	0.84
P579	20298901	262.777	0.00215	0.01813	1303.175	933.544	851.795	0.941	0.836
P580	29209101	206.927	0.0025	0.00232	1465.636	994.864	945.594	0.993	0.903
P581	55420662	235.373	0.00156	0.00185	2811.554	1719.364	1683.12	0.998	0.905
P582	32849831	252.719	0.00209	0.00311	2189.874	1422.084	1260.018	0.987	0.861
P583	22882356	235.914	0.00289	0.01494	1594.52	1044.756	945.032	0.97	0.879
P584	21965780	234.412	0.0026	1e-06	1537.724	1044.105	915.882	0.972	0.875
P585	55001891	310.683	0.00138	0.00206	3363.675	2391.188	1900.128	0.968	0.797
P586	25780370	277.416	0.0031	0.0026	1888.355	1220.563	921.153	0.907	0.918
P587	26528939	198.72	0.00344	0.00094	1605.514	1000.36	862.145	0.96	0.777
P588	24820063	252.759	0.0027	0.01155	1700.347	1114.972	1069.409	0.985	0.865
P589	59395311	190.925	0.00361	0.00336	2274.715	1249.815	1107.36	0.975	0.883
P590	27518162	234.533	0.0051	0.00225	1314.813	1003.477	963.295	0.988	0.756
P591	3716016	225.88	0.00619	1e-06	1405.238	1233.827	1214.68	0.997	0.77
P592	9026380	226.1	0.00387	1e-06	1876.617	1541.889	1242.198	0.999	0.764
P593	36666596	236.726	0.00354	0.01207	2044.155	1310.237	1180.904	0.981	0.869
P594	42852834	235.284	0.00436	0.0216	2267.848	1382.254	1246.38	0.984	0.869
P595	41298541	214.2	0.00286	0.04664	1517.622	1065.849	873.438	0.942	0.862
P596	41703370	241.274	0.00106	0.01128	2224.171	1367.338	1234.188	0.983	0.869
P597	28280720	230.736	0.0051	0.00176	1628.612	1162.172	1132.144	0.992	0.91
P598	33423335	232.364	0.00145	0.00047	1945.428	1412.008	1253.967	0.994	0.918
P599	23465953	260.064	0.00161	0.00122	1104.115	825.719	766.334	0.954	0.867
P600	55303457	240.858	0.00121	0.00222	3154.598	1978.306	1889.28	0.997	0.901
P601	55016608	199.2	0.00347	1e-06	2147.884	1429.007	1362.618	0.99	0.872
P602	21510523	253.515	0.00572	0.05503	1478.008	1142.525	1102.811	0.99	0.878
P603	57419503	247.801	0.00211	0.00056	3224.916	1925.785	1914.864	0.998	0.89
P604	25434516	252.677	0.00294	0.02454	1568.945	1104.37	1004.297	0.983	0.796
P605	48742865	254.185	0.00257	4e-04	2737.214	1753.884	1670.128	0.997	0.893
P606	39306043	235.764	0.00342	0.00849	2140.268	1341.188	1209.737	0.982	0.869
P607	44321814	193.397	0.00301	0.00188	3059.503	1642.666	1383.666	0.992	0.884
P608	40890384	205.817	0.0022	0.09964	2841.514	784.921	685.37	0.969	0.819
P609	23255053	261.193	0.00256	0.01378	1607.094	1151.764	1098.666	0.98	0.875
P610	32399826	266.399	0.00148	0.00047	1786.323	1291.868	1146.088	0.99	0.887
P611	23721690	248.804	0.00182	0.01704	1534.564	1073.123	984.923	0.965	0.852
P612	40723735	235.241	0.00389	0.00966	2191.373	1357.775	1224.033	0.983	0.869
P613	22213908	247.035	0.00382	0.00865	1520.651	1180.654	1093.464	0.99	0.877
P614	14850930	234.731	0.00328	0.02004	1255.9	1062.569	953.081	0.969	0.868
P615	16230914	240.163	0.00212	0.01167	1305.849	1080.683	967.657	0.97	0.869
P616	27807904	252.077	0.0027	0.0103	1917.666	1267.356	1200.376	0.99	0.869
P617	32856808	268.72	0.00124	0.00096	2096.402	965.032	969.47	0.992	0.803
P618	34345488	262.129	0.00146	0.00073	1897.428	1509.159	1130.706	0.989	0.891
P619	32945043	234.97	0.00232	0.00104	1858.079	1273.942	1243.769	0.994	0.9
P620	20338634	211.194	0.00314	0.01864	1300.605	867.449	752.411	0.932	0.83
P621	26093267	191.057	0.00223	0.0149	1871.759	1067.565	940.255	0.954	0.888
P622	50344717	194.106	0.00247	0.02279	3380.312	1673.727	1551.818	0.994	0.849
P623	44237443	211.996	0.00217	0.00071	2256.552	1418.164	1086.779	0.99	0.908
P624	14826051	238.654	0.00346	0.00065	993.403	866.427	585.704	0.903	0.862
P625	40196929	235.556	0.0029	0.00047	2270.526	1431.656	1402.187	0.997	0.893
P626	37920361	228.2	0.00275	0.03273	1354.976	938.629	823.736	0.957	0.867
P627	28692211	258.169	0.002	0.00042	1609.539	1262.117	1196.396	0.994	0.897
P628	47195559	236.563	0.00342	0.00981	2424.837	1430.674	1290.917	0.986	0.87
P629	22426645	251.572	0.00371	0.01456	1542.483	1031.491	909.331	0.963	0.869
P630	34455955	235.585	0.00293	0.00814	1964.425	1285.746	1158.718	0.98	0.869
P631	32990730	229.118	0.00387	0.00309	1906.627	1339.137	1261.116	0.993	0.91
P632	48419887	243.637	0.00381	0.10503	1707.121	1082.293	1028.973	0.982	0.86
P633	25326703	228.807	0.00483	1e-06	1753.134	1267.445	1264.549	0.997	0.88
P634	63453374	225.4	0.00294	0.05667	2251.574	1603.14	1496.982	0.995	0.863
P635	38401462	230.575	0.00545	0.0157	2106.296	1330.501	1199.617	0.982	0.869

P636	57104564	229.846	0.00132	0.00277	3213.137	1677.871	1594.498	0.998	0.888
